# School travel mode, parenting practices and physical activity among UK Year 5 and 6 children

**DOI:** 10.1186/1471-2458-14-370

**Published:** 2014-04-16

**Authors:** Russell Jago, Lesley Wood, Simon J Sebire, Mark J Edwards, Ben Davies, Kathryn Banfield, Kenneth R Fox, Janice L Thompson, Ashley R Cooper, Alan A Montgomery

**Affiliations:** 1 Centre for Exercise, Nutrition & Health Sciences, School for Policy Studies, University of Bristol, Bristol, UK; 2 School of Sport and Exercise Sciences, University of Birmingham, Birmingham, UK; 3 Nottingham Clinical Trials Unit, University of Nottingham, Nottingham, UK

**Keywords:** Physical activity, Travel, Parenting, Children, Adolescents

## Abstract

**Background:**

School travel mode and parenting practices have been associated with children’s physical activity (PA). The current study sought to examine whether PA parenting practices differ by school travel mode and whether school travel mode and PA parenting practices are associated with PA.

**Methods:**

469 children (aged 9-11) wore accelerometers from which mean weekday and after-school (3.30 to 8.30 pm) minutes of moderate-to-vigorous intensity PA (MVPA) and counts per minute (CPM) were derived. Mode of travel to and from school (passive *vs*. active) and PA parenting practices (maternal and paternal logistic support and modelling behaviour) were child-reported.

**Results:**

Children engaged in an average of 59.7 minutes of MVPA per weekday. Active travel to school by girls was associated with 5.9 more minutes of MVPA per day compared with those who travelled to school passively (p = 0.004). After-school CPM and MVPA did not differ by school travel mode. There was no evidence that physical activity parenting practices were associated with school travel mode.

**Conclusions:**

For girls, encouraging active travel to school is likely to be important for overall PA. Further formative research may be warranted to understand how both parental logistic support and active travel decisions are operationalized in families as a means of understanding how to promote increased PA among pre-adolescent children.

## Background

Physical activity (PA) is associated with lower levels of cardio-metabolic risk factors, higher levels of mental well-being and there is some evidence that PA is associated with a lower risk of obesity among young people [1]. National population-based surveys indicate that many children do not engage in the recommended 60 minutes of moderate-to-vigorous intensity PA (MVPA) every day [2-4]. PA levels differ by gender with girls typically being less active than boys at all ages [5]. PA declines during childhood, with the end of primary school and start of secondary school being an important period of change [5]. Strategies to increase PA at the end of primary school are therefore needed.

Systematic reviews have examined the degree of success of youth PA interventions [6-9]. The majority of interventions have been delivered during curriculum time, been of short duration, and yielded weak or modest effects. Some authors [10-12] have commented on the constraints of promoting PA during school hours because of the limited time that is available once provision has been made for “core” subjects (mathematics, English and science). The relative failure of curricular-time PA interventions implies that alternative intervention approaches may be needed [12]. The after-school period has been identified as “critical hours” [13] for young people’s PA, as it is perhaps the only period of the day during which young people can decide whether, and how, they are active. Patterns of PA during the after-school period have been shown to differ by socio-economic position with lower levels of participation in organised sports among children from lower income households [14]. Interventions to promote PA in the after-school period which can be provided for all socio-economic groups are therefore particularly worthy of attention [12,15].

Behaviour change is facilitated by identifying and modifying predictors of target behaviours such as PA [16,17]. To promote PA in the after-school period there is a need to identify modifiable factors that are associated with young people’s PA, both overall and particularly during the after-school period. Previous research has shown that children who walk home from school obtain MVPA from the journey and may also have higher levels of overall PA in the after-school period [18]. Thus, identifying the variables that are associated with school travel model is therefore important for identifying potential strategies to promote PA.

Recent studies have indicated that parents exert considerable influence on the PA patterns of children. Emerging evidence suggests that parental influence is a function of PA parenting practices (i.e. what a parent does to facilitate PA) rather than a direct association between parent and child PA [19-22]. In previous work, we have shown that the influence of PA parenting practices may differ by both parent and child sex, with paternal logistic support for PA being particularly important for PA among boys [20], and maternal support being more strongly associated with girls’ PA intention and self-efficacy than paternal support [23]. Previous work has also shown that active travel to school is a key source of PA for many children [24]. It is not currently clear, however, whether there are differences in the PA parenting practices of children who actively travel to school compared with their counterparts who are driven to school (passive travellers). This gap is important because the active travel of school children is ultimately arranged by parents. Some parents support and facilitate active travel while others do not and it is possible that any differences in travel mode could be related to PA parenting practices. Establishing whether there are any differences would therefore be an important first step in designing interventions.

In light of the evidence presented above, the aims of this paper were to examine, in this sample: 1) whether there was any difference in PA in those who travelled to school actively compared with those who travelled passively; 2) whether the PA practices of parents were associated with the travel mode of their children to and from school (active *vs*. passive); and 3) the extent to which mode of travel to and from school and PA parenting practices were associated with PA among young people. To facilitate the development of targeted approaches to the development of interventions we also examined whether associations were different for the PA accrued across the weekday (including activity that occurs before school) versus the activity accrued exclusively in the after-school period and whether associations were different for the type of PA (minutes of MVPA *vs*. volume of PA).

## Methods

### Sampling and participants

Data are from baseline assessments that were conducted as part of the Action 3:30 feasibility trial that examined the effect of an extracurricular physical activity programme on the PA levels of Year 5 and 6 pupils in October 2012 [15]. Pupils from years five and six were recruited from primary schools within Bristol, Bath and North-East Somerset (BANES), and South Gloucestershire. All 189 main stream state-funded primary schools, with the exception of 51 that were participating in concurrent studies, were invited to take part. Twenty schools were selected on a first-come-first-served basis. As participants were recruited and baseline data collected prior to randomisation all schools were limited to a maximum of 30 pupils per school as this was the maximum number of pupils that could be accommodated in the each session of the programme. Schools with fewer than 30 pupils in year 5 and year 6 combined were excluded. Where more than 30 pupils volunteered to participate, 30 were randomly selected using a simple random selection procedure. The study received ethical approval from the School for Policy Studies Research Ethics Committee at the University of Bristol (ref: Action 3:30 Project) and written parental consent was obtained for all participants.

### Measures

#### Travel mode to and from school

Children reported how they usually travelled to and from school. For each journey children chose from four options: walk, cycle or scoot, car, bus/train. Walking and cycling/scooting were re-coded as active travel whilst car and public transport were classified as passive travel. Travel to and from school were examined separately as some children used different modes of travel in each direction.

#### Parent PA support

Parental support for PA was measured using a modified version of the Revised Parent Activity Support Scale – Child Report Version [25-27]. The parent support scale provides a global assessment of modelling (the extent to which the parent models an active lifestyle) and logistic support (the parent facilitates PA) for the child. Eight items from the original scale, representing modelling and logistic support, were used with five items assessing parental modelling and three representing logistic support. Each item required a response on a four-point scale (from ‘Disagree a lot’ to ‘Agree a lot’). The eight items were repeated with reference to the mother (or step-mother) and father (or step-father) to give up to four child-perceived subscales (i.e. maternal and paternal logistic support, and maternal and paternal modelling). Examples of the statements to which children were asked to respond include “My mum often exercises or does something active” (modelling) and “My mum watches me compete in sporting events or other physical activities” (logistic support). Cronbach’s alpha was computed for each subscale and varied from 0.85 (paternal modelling) to 0.62 (maternal logistic support). For both subscales, internal consistency was higher for the paternal versions than for the equivalent maternal versions.

#### Height and weight

Child height was measured using a SECA Leicester stadiometer (HAB International, Northampton). Weight was recorded using a SECA 899 digital scale (HAB International, Northampton). Body mass index (BMI = kg/m^2^) was calculated and converted to an age and gender specific standard deviation score (BMI z-score) based on 1990 UK child growth reference curves [28] using the Stata function ‘zanthro’ command [29].

#### Deprivation

An index of multiple deprivation (IMD) score, using the English Indices of Deprivation (http://data.gov.uk/dataset/index-of-multiple-deprivation), was assigned to each participant based on their home postcode linked to the respective lower layer super output area (LLSOA). The IMD score estimates area deprivation based on several indicators chosen to cover a range of economic, social and housing criteria, which are combined into a single deprivation score for each small area in England. The 32,482 LLSOAs in England have a range of 0.53 to 87.8 and a median of 17.25 with a higher score indicating a greater level of deprivation.

#### Physical activity

PA was assessed using an Actigraph accelerometer (Model GT3X+; ActiGraph LLC, FL, USA) which was set to collect data at 30 Hz for a maximum of five days including a weekend, to provide a maximum of three weekdays. Participants were instructed to remove the monitor for sleeping and bathing. Actigraph accelerometers have been shown to provide estimates of energy expenditure that are closely associated with laboratory derived energy expenditure [30]. Periods of ≥60 minutes of zero values, with an allowance of up to two minutes of interruptions, were defined as accelerometer “non-wear” time and were removed from the analyses [4]. To maximise the study sample participants were included if they had provided at least two weekdays of valid accelerometer data (a valid day was defined as the provision of at least 500 minutes of data between 6 am and 11 pm). We adopted this approach because previous research has shown no difference in the reliability of accelerometer data if 420 or 600 minutes was used as a criterion for a valid day of data [31]. As such, 500 minutes represented a pragmatic approach to processing the accelerometer data that is consistent with previous studies [32] while ensuring that the sample was as large as possible. An after-school window was also created to detect PA that occurred between the end of school (3:30) and evening (8.30 pm). The 8:30 pm cut-off was used as previous global positioning system data conducted in Bristol has shown that very little PA occurs outside after 8:30 pm and as such the 3:30 to 8:30 pm window provided a large window in which to capture activity after-school including the journey home from school [33]. Mean minutes of MVPA on a weekday and in the after-school period were derived using a cut-point of ≥2296 counts per minute [34]. Mean counts per minute (CPM) per day and during the after-school period on weekdays were also determined to provide an estimate of PA volume.

#### Distance from the home address to the school

The distance between each pupil’s home address and their school was calculated using the straight line difference between the geographic Cartesian coordinates (based on postcodes) of the two addresses.

### Statistical analysis

Descriptive statistics, including means, standard deviations, frequencies and percentages, were calculated for variables as appropriate. Student t-tests were used to examine differences between genders for continuous measures (i.e., age, BMI z-score, IMD, subscales of the Activity Support Scale, and objective PA measures). Associations between travel mode (both to school and home from school) and child gender were examined using logistic regression models. Student t-tests were also used to identify any potential bias due to differences in the BMI or socio-economic status of participants who provided valid accelerometer data when compared to those who did not meet the accelerometer inclusion criteria. Of the 469 included participants, 24 (5.1%) provided valid data for two days; 96 (20.5) provided it for three days; 137 (29.2) for four days; 212 (45.2) for five days. Seventy children did not meet the inclusion criteria, due either to insufficient accelerometer data or incomplete information for variables used to adjust the analysis.

Multi-variable logistic regression models were used to examine whether distance from home to school or parenting practices were associated with the method of travel to and from school. Models were adjusted for IMD and, since the children were recruited from schools, each analysis was also adjusted for within-school clustering by the use of robust standard errors. These correct the standard errors by taking account of the similarity of individuals within the same cluster and are important because presence of clustering tends to estimate artificially small standard errors which in turn increases the risk of a type I error [35]. Linear regression models were subsequently used to further investigate the extent to which travel mode to and from school, distance from school, and parental support for PA predicted four separate elements of children’s PA (counts per minute and minutes of MVPA, for weekdays and during the after-school period on weekdays). These models were adjusted for household IMD, BMI z-score and within-school clustering. To test whether the relationship between the exposures and outcomes differed by gender within each model, the interaction between each exposure and child gender was tested. Taking the results of the interactions, together with the fact that children’s PA has been shown to differ by gender [35], there was sufficient evidence to stratify each analysis by gender. Results are therefore shown for girls and boys separately. The adjusted R^2^ is shown for the linear regression models as an estimate of the amount of variance in the dependent variable explained by the overall model. It is important to note that the estimate is specific to the data under test and is not generalizable [36]. In light of the number of different analyses that were conducted, an arbitrary p-value indicating ‘statistical significance’ was not set but rather analyses were interpreted using point estimates and 95% confidence intervals with decreasing p-values indicating increasing strength of evidence against the null hypothesis [37]. All analyses were performed in Stata version 12.0 (College Station, Texas).

## Results

Descriptive statistics are shown by gender in Table 1. The 469 included participants had a mean age of 10.0 years, with ages ranging from 9.1 to 11.1. Children in the sample had an average of 59.7 minutes of MVPA per day (data not shown), with boys engaging in 69 minutes and girls engaging in 53 minutes per day indicating that the children in this sample were relatively active. There was strong evidence that boys engaged in higher levels of MVPA overall and after-school and a higher volume of PA (CPM) overall when compared to girls. There was also some evidence of a higher volume of activity, measured by CPM, in boys compared with girls during the after-school period. There was some evidence that boys had higher paternal PA modelling and paternal logistic support than girls but the magnitude of differences was small (0.2 and 0.3 respectively). There was also good statistical evidence that a greater proportion of boys than girls actively travelled home from school (70.2% vs. 57.8%). Overall, 65 pupils (13.9%) reported that they cycled to school, with 66 (14.1%) cycling on the return journey. Fifty-nine pupils (13%) reported cycling in both directions with a further 209 (45%) saying that they walked in both directions. Among the passive travellers 156 pupils travelled to school by car and only three by bus or train. For the journey home 168 pupils travelled by car, with five using a bus or train.

**Table 1:** Descriptive statistics of all variables used in the subsequent analyses for participants by gender

	**Boys (n = 201/197**^†^/**191**^††^)		**Girls (n = 268/263**^†^/**250**^††^)				
	**Mean**	**SD**	**Mean**	**SD**	**Difference in means**	**95% CI**	**p**
Age (years)	10.0	0.6	10.1	0.6	-0.04	-0.14 to 0.06	0.445
BMI (kg/m^2^)	18.5	3.2	18.6	3.4	-0.07	-0.68 to 0.54	0.819
BMI z-score*	0.7	1.1	0.4	1.2	0.3	0.05 to 0.47	0.016
IMD score (high is more deprived)^†^	20.5	17.1	19.8	17.8	0.8	-2.46 to 4.03	0.634
Distance to school (miles)^††^	0.58	0.68	0.58	0.58	0.008	-0.11 to 0.12	0.894
**Physical activity**							
* Weekdays*							
CPM	603.3	166.9	507.1	121.2	96.1	70.00 to 122.28	<0.001
MVPA (mins)	68.6	26.1	53.0	16.1	15.7	11.81 to 19.50	<0.001
After school weekdays**							
CPM	620.6	380.3	549.2	292.4	71.4	10.35 to 132.42	0.022
MVPA (mins)	14.3	9.0	11.6	5.8	2.6	1.28 to 3.98	<0.001
**Parental support for PA**	n = 190/186^‡^		n = 249/ 237^‡^				
Maternal modelling	2.9	0.8	2.9	0.7	0.02	-0.12 to 0.16	0.786
Maternal logistic support	3.3	0.7	3.2	0.7	0.06	-0.07 to 0.19	0.389
Paternal modelling^‡^	3.2	0.8	3.1	0.8	0.2	0.003 to 0.29	0.045
Paternal logistic support^‡^	3.3	0.8	3.0	0.9	0.3	0.13 to 0.44	<0.001
	**Boys**		**Girls**				
Travel to school (n = 469)	**n**	**%**	**n**	**%**	**OR**	**95% CI**	**p**
Passive (reference group)	63	31.3	96	35.8	0.82	0.55 to 1.20	0.311
Active	138	68.7	172	64.2			
Travel home (n = 469)							
Passive (reference group)	60	29.9	113	42.2	0.58	0.40 to 0.86	0.006
Active	141	70.2	155	57.8			

*Based on 1990 UK growth charts.

**After school period is from 3.30 pm to 8.30 pm.

^†^Number providing information for IMD.

^††^Number providing information for distance to school.

^‡^Number providing information for paternal support variables.

Although there was no difference in the age-adjusted BMI scores of those who provided valid accelerometer data when compared to those that did not meet the accelerometer inclusion criteria (difference in means: 0.02; 95% CI: -0.32 to 0.36; p = 0.905), there was evidence that excluded participants had higher IMD scores than those with valid accelerometer data (25.0 *vs*. 20.1; difference in means: 4.9; 95% CI: 0.50 to 9.4; p < 0.001) indicating that participants with valid data were more likely to come from higher socio-economic groups.

Table 2 summarizes PA by school travel mode. There was no evidence of a difference in CPM or MVPA on weekdays overall or in the after-school period of boys who were active travellers (both to and from school) when compared with those who were passive travellers. A uni-variable analysis indicated that girls who actively travelled to school obtained an average of 5.9 more minutes of MVPA per day (95% CI 1.9 to 9.9, p = 0.004) compared with those who travelled passively, with no differences between girls who travelled actively compared with those who travelled passively on the way home. The increased PA of girls who travelled actively on the way to school was supported to some extent by the weaker evidence that this group had a higher volume of PA, indicated by higher CPM (517 in active travellers compared with 490 in girls who were passive travellers; p = 0.077). There was, however, a lack of evidence of any difference in after-school activity between the two groups of girls.

**Table 2:** **Uni**-**variable analyses** (**showing mean and sd**) **of physical activity and distance to school by travel mode to and from school**

**Travel to school**										
	**Boys**					**Girls**				
	**Passive n = 63**	**Active n = 138**	**Difference in means**	**95% CI**	**p**	**Passive n = 96**	**Active n = 172**	**Difference in means**	**95% CI**	**p**
**Weekday CPM**	581.7 (173.9)	613.1 (163.3)	-31.4	-81.35 to 18.59	0.217	489.6 (115.8)	516.9 (123.4)	-27.3	-57.61 to 2.94	0.077
**Weekday MVPA (mins)**	64.9 (26.9)	70.3 (25.7)	-5.4	-13.23 to 2.39	0.173	49.2 (14.9)	55.1 (16.4)	-5.9	-9.89 to -1.93	0.004
**After-school CPM**	664.2 (419.4)	600.7 (360.9)	63.4	-50.55 to 177.40	0.274	517.5 (263.6)	566.9 (306.6)	-49.5	-122.7 to 23.8	0.185
**After-school MVPA (mins)**	15.1 (12.3)	13.9 (7.8)	1.2	-1.50 to 3.90	0.384	10.9 (5.2)	12.1 (6.1)	-1.2	-2.66 to 0.25	0.104
**Distance to school (miles)**	0.98 (0.86)	0.41 (0.49)	0.57	0.38 to 0.77	<0.001	0.98 (0.77)	0.36 (0.26)	0.62	0.49 to 0.75	<0.001
**Travel home from school**										
	**Boys**					**Girls**				
	**Passive n = 60**	**Active n = 141**	**Difference in means**	**95% CI**	**p**	**Passive n = 113**	**Active n = 155**	**Difference in means**	**95% CI**	**p**
**Weekday CPM**	573.0 (169.0)	616.2 (164.9)	-43.1	-93.61 to 7.38	0.094	507.2 (117.8)	507.1 (124.0)	0.05	-29.52 to 29.62	0.998
**Weekday MVPA (mins)**	63.8 (27.8)	70.7 (25.2)	-6.8	-14.74 to 1.05	0.089	51.7 (16.1)	53.8 (16.1)	-2.1	-6.0 to -1.8	0.292
**After-school CPM**	633.4 (373.3)	615.2 (360.9)	18.2	-97.69 to 134.03	0.757	536.6 (279.1)	558.4 (302.3)	-21.8	-93.1 to 49.5	0.548
**After-school MVPA (mins)**	14.4 (10.6)	14.2 (8.3)	0.18	-2.56 to 2.93	0.894	11.3 (5.5)	11.9 (6.1)	-0.54	-1.96 to 0.88	0.455
**Distance to school (miles)**	1.0 (0.88)	0.41 (0.68)	0.60	0.40 to 0.79	<0.001	0.92 (0.73)	0.33 (0.24)	0.59	0.46 to 0.72	<0.001

Results from logistic regression models suggest that the only predictor of travel mode for both genders was the distance from home to school. There was no evidence that the parenting practices of either mothers or fathers were important determinants for the method of travel (Table 3).

**Table 3:** **Multi**-**variable logistic regression models predicting travel mode to and from school by parenting practices and distance between home and school**

Travel to school*						
	**Boys (n passive: 56; n active: 117)**			**Girls (n passive: 77; n active: 138)**		
	**OR**	**95% CI**	**p**	**OR**	**95% CI**	**p**
**Maternal modelling**	1.15	0.70 to 1.91	0.581	1.12	0.52 to 2.39	0.768
**Maternal logistic support**	1.06	0.57 to 1.97	0.852	0.92	0.48 to 1.78	0.812
**Paternal modelling**	0.75	0.40 to 1.40	0.372	0.84	0.48 to 1.49	0.560
**Paternal logistic support**	1.36	0.73 to 2.51	0.330	1.29	0.78 to 2.14	0.328
**Distance to school (miles)**	0.11	0.03 to 0.43	0.002	0.03	0.01 to 0.10	<0.001
**Model statistics**	**Pseudo R**^**2**^**: 0.206, p** **=** **0.008**				**Pseudo R**^**2**^**: 0.274, p** < **0.001**	
	Travel home from school*					
	**Boys (n passive: 55; n active: 118)**			**Girls (n passive: 89; n active: 126)**		
	**OR**	**95% CI**	**p**	**OR**	**95% CI**	**p**
**Maternal modelling**	1.10	0.60 to 2.01	0.767	0.87	0.43 to 1.79	0.713
**Maternal logistic support**	1.01	0.49 to 2.06	0.978	1.16	0.69 to 1.93	0.579
**Paternal modelling**	1.13	0.71 to 1.78	0.612	0.93	0.45 to 1.92	0.846
**Paternal logistic support**	1.33	0.66 to 2.64	0.424	1.12	0.63 to 2.02	0.693
**Distance to school (miles)**	0.12	0.03 to 0.42	0.001	0.03	0.004 to 0.19	<0.001
	**Pseudo R**^2^**: 0.198, p = 0.011**			**Pseudo R**^2^**: 0.254, p < 0.001**		

*Reference category: passive travel.

All models are adjusted for IMD and within-school clustering.

Linear regression models investigating the associations between weekday physical activity and modelling/support, travel mode, and distance from home to school are shown by gender in Table 4. After adjusting for IMD and BMI-z score, there was a lack of evidence that any of the potential explanatory variables could predict PA in boys. For girls, the fully adjusted model suggested that there was some evidence that active travel to school was associated with 6.5 more minutes of physical activity when compared to passive travel (p = 0.034). For the after-school period there was no evidence that parenting practices or school travel model, either alone or in combination for the whole model, predicted either of the PA variables (CPM or MVPA Table 5).

**Table 4:** **Linear regression models predicting weekday physical activity from PA parenting practices**, **distance to school and travel mode to and from school**

	**Boys (n** **=** **173)**					
	**CPM**			**MVPA (mins)**		
	**Coeff**	**95% CI**	**p**	**Coeff**	**95% CI**	**p**
**Maternal modelling**	16.67	-24.16 to 57.51	0.403	1.73	-5.18 to 8.65	0.606
**Maternal logistic support**	-21.55	-75.10 to 32.00	0.410	-2.46	-11.85 to 6.93	0.590
**Paternal modelling**	15.46	-22.85 to 53.77	0.409	0.50	-6.23 to 7.23	0.879
**Paternal logistic support**	27.60	-12.55 to 67.76	0.167	5.52	-0.28 to 11.33	0.061
**Distance to school (miles)**	-7.76	-46.09 to 30.56	0.676	-0.85	-7.14 to 5.45	0.781
**Travel to school (ref** **=** **Passive)**	-18.14	-120.30 to 84.02	0.714	-2.77	-21.02 to 15.49	0.754
**Travel home (ref** **=** **Passive)**	25.00	-457.72 to 107.72	0.676	3.72	-11.32 to 18.76	0.611
**Model statistics**	**Adjusted R**^**2**^**: 0.165, p** **=** **0.001**			**Adjusted R**^**2**^**: 0.188, p** < **0.001**		
	**Girls (n** **=** **215)**					
	**CPM**			**MVPA (mins)**		
	**Coeff**	**95% CI**	**p**	**Coeff**	**95% CI**	**p**
**Maternal modelling**	9.40	-18.53 to 37.33	0.490	0.28	-4.45 to 5.01	0.903
**Maternal logistic support**	18.49	-9.05 to 46.02	0.176	2.70	-1.44 to 6.83	0.188
**Paternal modelling**	-1.22	-29.27 to 26.82	0.928	-1.05	-4.96 to 2.85	0.579
**Paternal logistic support**	-7.31	-37.72 to 23.10	0.621	0.41	-3.79 to 4.60	0.841
**Distance to school (miles)**	-11.70	-47.73 to 24.34	0.505	-1.21	-5.38 to 3.00	0.550
**Travel to school (ref** **=** **Passive)**	34.76	-9.87 to 79.39	0.120	6.52	0.53 to 12.51	0.034
**Travel home (ref** **=** **Passive)**	-26.05	-69.52 to 17.41	0.225	-3.53	-9.66 to 2.60	0.243
**Model statistics**	**Adjusted R**^2^**: 0.042, p = 0. 014**			**Adjusted R**^2^**: 0.053, p = 0. 002**		

All models are adjusted for IMD, BMI z-score, and within-school clustering.

**Table 5:** **Linear regression models predicting weekday physical activity in the after**-**school period**

	**Boys (n = 173)**					
	**CPM**			**MVPA**		
	**Coeff**	**95% CI**	**p**	**Coeff**	**95% CI**	**p**
**Maternal modelling**	61.91	-46.89 to 170.71	0.248	1.27	-0.68 to 3.22	0.190
**Maternal logistic support**	-41.96	-148.68 to 64.75	0.421	-1.20	-4.59 to 2.20	0.470
**Paternal modelling**	42.21	-23.93 to 108.35	0.197	1.63	-0.91 to 4.16	0.196
**Paternal logistic support**	-19.78	-112.78 to 73.22	0.661	0.37	-1.98 to 2.72	0.745
**Distance to school (miles)**	56.38	-96.39 to 209.16	0.449	-0.56	-0.56 to 1.22	0.651
**Travel to school (ref = passive)**	32.32	-172.55 to 237.18	0.745	-2.64	-11.07 to 5.78	0.519
**Travel home (ref = passive)**	-48.99	-179.16 to 81.18	0.441	0.68	-5.44 to 6.79	0.819
**Model statistics**	**Adjusted R**^**2**^**: 0.069; p = 0.004**			**Adjusted R**^**2**^**: 0.103; p = 0.024**		
	**Girls (n = 215)**					
	**CPM**			**MVPA**		
	**Coeff**	**95% CI**	**p**	**Coeff**	**95% CI**	**p**
**Maternal modelling**	-12.53	-71.82 to 46.76	0.663	-0.01	-1.44 to 1.43	0.991
**Maternal logistic support**	46.62	-1.16 to 94.40	0.055	0.55	-1.36 to 2.47	0.554
**Paternal modelling**	-28.11	-83.90 to 27.68	0.305	-0.83	-2.28 to 0.62	0.248
**Paternal logistic support**	33.54	-29.67 to 96.75	0.281	0.65	-1.32 to 2.61	0.498
**Distance to school (miles)**	4.00	-87.12 to 95.06	0.928	-0.59	-1.62 to 0.43	0.243
**Travel to school (ref = Passive)**	65.85	-99.87 to 231.56	0.416	0.50	-1.75 to 2.75	0.648
**Travel home (ref = Passive)**	-19.83	-136.31 to 96.66	0.726	-0.20	-2.25 to 1.84	0.836
**Model statistics**	**Adjusted R**^2^**: 0.067;** p **=** 0.133			**Adjusted R**^2^**:** 0.040**;** p **=** 0.289		

All models are adjusted for IMD, BMI z-score, and within-school clustering.

## Discussion

The data presented in this paper have shown that for 9 to 11 year old girls, active travel to school was associated with an average of six additional minutes of MVPA per day. There was some evidence that active travel to school by girls was also associated with a higher volume of PA as indicated by higher accelerometer counts per minute. For boys, there was little evidence that active travel was associated with higher levels of MVPA or CPM. There was no difference in the proportion of boys and girls who were active travellers to school, although boys were more likely than girls to be active travellers on the way home from school. Consistent with previous research [5,38] there was strong evidence that boys engaged in a higher number of minutes of MVPA on a weekday than girls, which may indicate that for these less active girls, active travel is a particularly important source of PA. As previous research has reported that at the end of primary school boys have higher levels of independent mobility to be active in their local neighbourhood than girls [39], strategies that focus on promoting active travel for pre-adolescent girls are warranted.

The analyses presented in this paper do not appear to indicate that paternal or maternal parenting practices were different for children who engaged in active or passive modes of travel to school. These analyses were performed based on the hypothesis that parents who provided either more modelling or logistic support for PA may also be more likely to facilitate active travel as part of the overall supportive approach to PA within the household. The logistic support scale assesses practical support to facilitate participation in PA but does not assess active travel. Thus, the absence of any evidence of an association suggests that logistic support for PA and active travel are unlikely to be related. As these scales have been previously used in UK samples and been shown to be associated with overall PA [19,23], it appears that the absence of an association is unlikely to be a function of measurement error. Further formative research may be warranted to understand how both parental logistic support and active travel decisions are operationalized in families as a means of understanding how to promote increased PA among pre-adolescent children. Once such work has been completed a scale that specifically assesses active travel parenting practices could be developed and such a scale would be of great use to researchers in the field.

There was no evidence that active travel from school was associated with higher levels of MVPA or CPM in the after-school period. This lack of an association is surprising as logically any effect of active travel would be most likely to occur in the after-school period when the active travel occurs. Moreover, previous studies including research conducted in Bristol [40,41] have shown that active travel from school is associated with higher levels of MVPA in the after-school period. This disagreement between our findings and previous work is hard to reconcile but could be a function of the sample as the participants included in this study had expressed an interest in attending an extra-curricular (after-school) PA programme. Furthermore, the combined sample obtained an average of 59.7 minutes of MVPA at baseline indicating that this group of children were a group of active children who were close to meeting public health guidance. It may therefore be the case that this sample included a more active group of children who were more likely to attend organised forms of PA and these factors could have attenuated differences between the active and passive travellers in the after-school period.

In the current analyses children who adopted passive models of travel to school resided further away from school than active travellers. Distance to school remained the strongest predictor of mode of travel to school in the multivariable models. These associations are consistent with previous research which has shown that greater proximity to school is associated with increased walking to school [42,43]. Thus, it may be the case that any possible impact of parenting practices on travel to school is greatly attenuated by the distance from school.

In this study we found that a greater proportion of boys adopted active modes of travel home from school than girls (70% vs. 58%). It is noticeable that travel patterns to school were broadly comparable between the genders with 68% of boys and 64% of girls adopting active travel to school. This might suggest that in this sample there is a specific difference in the mode of travel home from school. Comparing the data from this study to other studies is complex as the majority of studies in this area report usual mode of travel to school [39,44]. In studies that have examined gender differences in mode of travel to school there have been some studies reporting that that boys are more likely to adopt active modes of travel to school than girls [45] while other studies have reported no gender differences [46]. Thus, when our findings are viewed in relation to the wider literature, current evidence suggests that there is a need to examine in different datasets, whether there is an interaction between gender and mode of travel to and from school. Furthermore, if there is evidence that associations are different there is a need to examine which factors are contributing to this difference.

### Strengths and limitations

The major strengths of this paper are the assessment of PA parenting practices, active travel and accelerometer assessed PA among UK children. It is important to recognise that stratifying the sample for gender specific analyses limited the power to detect some associations and this limitation is highlighted by the wide 95% confidence intervals for the parenting variables. We used the child reported Parent Activity Support Scale which measures global physical activity parenting practices. This measure does not specifically assess active travel and it may therefore be the case that there is a need for an active travel specific parenting measure. As accelerometers are worn on the hip they do not provide accurate assessments of time spent cycling. As such the overall levels of physical activity among cyclists is likely to have been underestimated, which is likely to have limited our ability to detect differences between active and passive travellers. This limitation may be particularly important for this study as the study focussed on active travel. It may be possible to overcome such issues in the future by asking participants to log the time that cycling occurs and align these data with the accelerometer data but such an approach would be difficult as children struggle to recall accurately when events occur.

As noted above, the sample was drawn from the baseline data collection of a randomised controlled feasibility trial that focussed on the promotion of PA via extra-curricular PA clubs, and, consequently, participants might have a greater inclination towards participation in after-school clubs. The data have also shown that the boys engaged in an average of 68.6 minutes of MVPA with 53.0 minutes for the girls which suggests that the participants were an active group of children and as such the representativeness of this group may be limited. Moreover, as data were collected from schools in a largely urban part of the UK we were unable to examine differences between participants residing in urban versus rural areas. Finally, due to the cross-sectional nature of the data it is not possible to infer causation in the relationships between parenting practices, active travel and PA.

## Conclusion

There was no evidence of differences in the PA parenting practices of children who have active modes of travelling to school when compared to children who have passive modes of school travel. For girls, active travel to school was associated with an additional six minutes of MVPA per weekday and as such, encouraging active travel to school is likely to be important for overall PA among girls.

## Competing interest

The authors declare that they have no competing interests.

## Authors’ contributions

The study was conceived by RJ, SJS and LW. Funding was secured by RJ, SJS, ARC, JLT, KRF and AAM. MJE and BD were the project managers and KB led the data collection. Data analysis was performed by LW with critical guidance from AAM. RJ produced the first draft of the paper with all other authors providing sections and critically reviewing the paper. All authors approved submission.

## Pre-publication history

The pre-publication history for this paper can be accessed here:

http://www.biomedcentral.com/1471-2458/14/370/prepub

## Data sharing

Data from the Action 3:30 study are not available to external collaborators until September 2015. From this point we would be happy for external collaborators to access these data according to data transfer agreements that will have been developed by then. Information regarding this access will be made available on the study website (http://www.bristol.ac.uk/sps/research/researchprojectpages/action330/index.html).
